# Multidisciplinary approach to rare primary cardiac sarcoma: a case report and review

**DOI:** 10.1186/s12885-019-5705-2

**Published:** 2019-05-31

**Authors:** Audrone Vaitiekiene, Domas Vaitiekus, Laura Urbonaite, Antanas Jankauskas, Justina Portacenko, Tomas Lapinskas, Rimantas Benetis, Adakrius Siudikas, Audrone Veikutiene, Lina Poskiene, Ausra Kavoliuniene, Rasa Janciauskiene, Laimonas Jarusevicius, Elona Juozaityte, Remigijus Zaliunas, Egle Ereminiene

**Affiliations:** 1 Department of Cardiology, Medical Academy, Lithuanian University of Health Sciences, Kaunas, Lithuania; 2 Department of Oncology and Hematology, Medical Academy, Lithuanian University of Health Sciences, Kaunas, Lithuania; 3 Department of Radiology, Medical Academy, Lithuanian University of Health Sciences, Kaunas, Lithuania; 4 Department of Cardiac, Thoracic and Vascular Surgery, Medical Academy, Lithuanian University of Health Sciences, Kaunas, Lithuania; 5 Institute of Cardiology, Lithuanian University of Health Sciences, Kaunas, Lithuania; 6 Department of Pathology, Medical Academy, Lithuanian University of Health Sciences, Kaunas, Lithuania

**Keywords:** Cardiac sarcoma, Cardiovascular magnetic resonance, Undifferentiated pleomorphic sarcoma

## Abstract

**Background:**

Undifferentiated pleomorphic sarcoma is a very rare and aggressive type of primary cardiac tumors. Most cardiac sarcomas result in rapid growth and quick death. According to different sources the median survival is typically 6 to 12 months. We are presenting a case of primary cardiac sarcoma with 26 months disease free survival following cytoreductive surgery and chemotherapy.

**Case presentation:**

A 48-year-old woman with progressing symptoms of dyspnea and palpitations for over 2 months was referred to a cardiologist. With the help of echocardiography and cardiovascular magnetic resonance cardiac sarcoma was suspected. Open biopsy and cytoreductive surgery were performed, complete resection of the tumor was not possible. Histology revealed undifferentiated pleomorphic sarcoma. Seven cycles of chemotherapy with Doxorubicine and Ifosfamide were completed. Cardiovascular magnetic resonance revealed a complete response – only signs of fibrosis without any signs of tumor were visible. Follow ups with echocardiography, cardiovascular magnetic resonance and chest, abdomen and pelvic computed tomography is performed every 3 months. Twenty-six months from initial diagnosis the patient is still free of recurrence of tumor with no compromises of the quality of life.

**Conclusion:**

Standard chemotherapy together with cytoreductive surgery can have a complete response effect in undifferentiated pleomorphic sarcoma with unusual long-term survival.

## Background

Undifferentiated pleomorphic sarcoma (UPS), also known as malignant fibrous histiocytoma is an extremely rare type of primary cardiac tumor [1, 2]. According to a study conducted in Cleveland Clinic UPS were diagnosed in 12% of all cardiac sarcoma cases [3]. Clinical presentation varies, they can be symptomatic, imitating different cardiac conditions (symptoms depend on the localization of tumor, invasion into the myocardium, but not histology) or found incidentally on echocardiography, MRI or CT examinations. UPSs are aggressive and locally invasive tumors, frequently making complete surgical excision unfeasible, which leads to a poor prognosis and a low survival rate [4]. Even with complete resection, most patients develop recurrent disease rapidly, thus according to different sources the median survival is typically 6 to 12 months [5]. We are presenting a case of primary cardiac sarcoma with 26 months disease free survival following cytoreductive surgery and chemotherapy.

## Case presentation

A 48-year-old female with progressing symptoms of dyspnea and palpitations for over 2 months was referred to cardiologist. Her medical history was unremarkable, she only had allergy to various chemical substances as she was working as a tailor. Auscultation of the heart revealed a loud systolic murmur throughout entire precordium with irradiation to the left shoulder blade. Hemodynamic parameters were normal, while the ECG showed left ventricular hypertrophy (LVH). Laboratory findings revealed a normocytic normochromic anemia (Hb 104 g/l, normal values 119-146 g/l) and elevated level of lactate dehydrogenase (LDH) without liver or renal dysfunction.

Transthoracic echocardiography (TTE) was performed. Several large masses in the left ventricle (LV) close to anterior and anterolateral mid-ventricular and apical segments were observed with one tumor (approximately 2.0 cm in length) partially obstructing LV outflow tract. Additional smaller tumor was seen in left atrium (LA) attached to the interatrial septum (IAS).

**Fig. 1:**
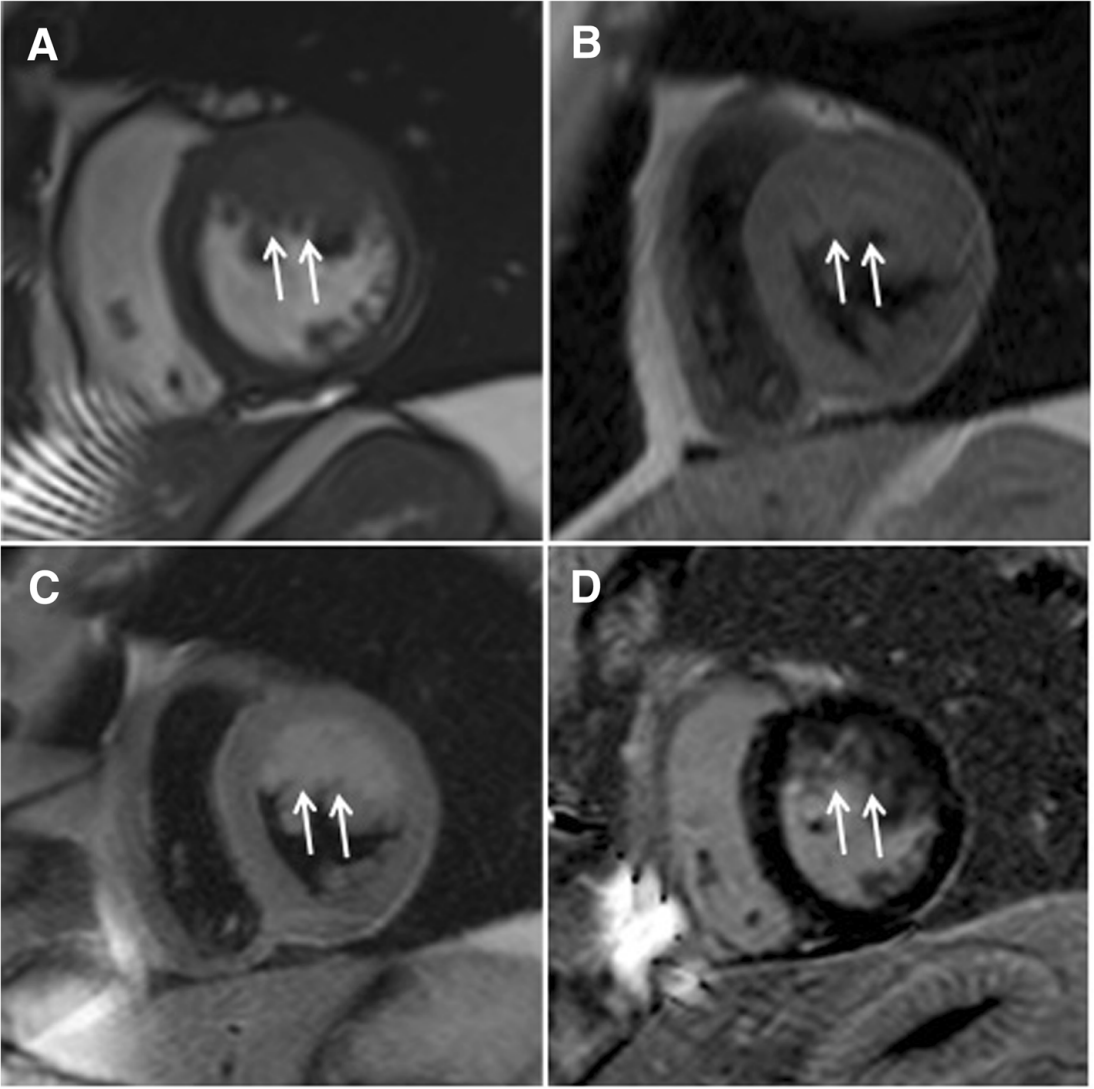
CMR examination before surgery. The cine image (**a**) demonstrates tumor (arrows) in the LV adherent to the anterior and anterolateral walls. Mass (arrows) appears isointense in T1-weighted (**b**), hyperintense in T2-weighted (**c**) and heterogeneously enhanced in late gadolinium enhancement (**d**) images

Cardiovascular magnetic resonance (CMR) was performed to assess specific characteristics of masses using a 1.5 T scanner (Siemens Magnetom Aera, Siemens AG Healthcare Sector, Erlangen, Germany) with an 18-channel phased array coil. Cine images (Fig. 1a) showed a 71 × 45 × 21 mm tumor with irregular borders in the LV attached to anterior and anterolateral walls. The mass was partially infiltrating LV myocardium and was isointense on non-contrast T1W spin-echo images (Fig. 1b). On T2W spin-echo images the tumor appeared hyperintense (Fig. 1c), early gadolinium enhancement was similar to myocardium. The tumor heterogeneously enhanced after administration of full dose of contrast agent (Fig. 1d). The diagnosis of malignant cardiac sarcoma was suspected. Also computed tomography (CT) of the chest and abdomen was performed, but there were no signs of other origin of malignancy or metastasis.

**Fig. 2:**
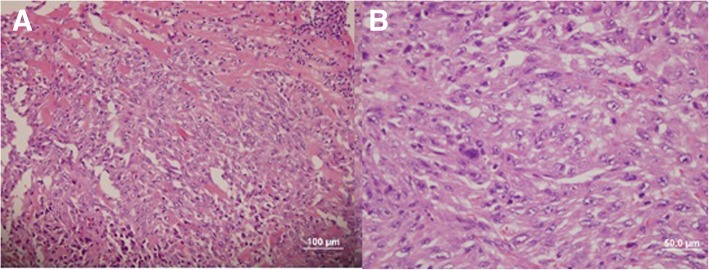
Histological examination: undifferentiated pleomorphic sarcoma: **a** – infiltration among cardiomyocytes, **b** – cell morphology

Open biopsy and cytoreductive surgery were performed. The masses from LV cavity, outflow tract and LA were removed, apical trabeculae were infiltrated by the tumor and complete resection was not possible. Histology revealed UPS. (Fig. 2) Cardiomyocytes were infiltrated with tumor cells. The tumor was composed of pleomorphic cells with abundant mitoses and area of necrosis. Focal positivity of CD34, CD99 and TLE-1 were not specific. Tumor cells were negative for actin, desmin, CD34, CD117, S100P, EMA and panCK immunomarkers.

**Fig. 3:**
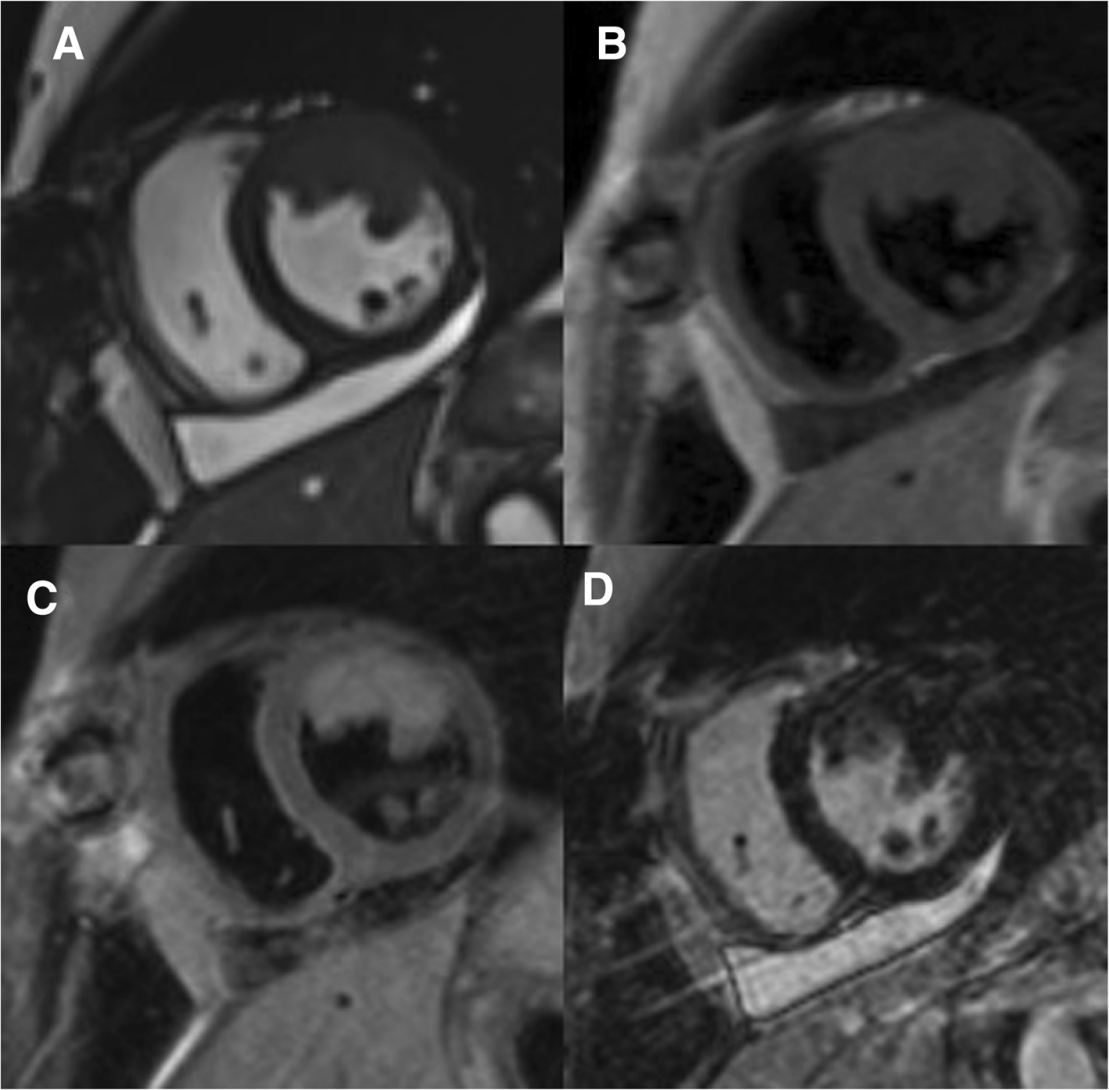
Residual tumor masses after cytoreductive surgery in the anterior and lateral wall of LV in cine (**a**), T1-weighted (**b**), T2-weighted (**c**) and LGE (**d**) images

Postoperative CMR revealed no masses in the LA and LV outflow tract. Tumor related to the LV cavity was still present (Fig. 3). Additionally, there was a pericardial effusion of 12 mm localized at LV inferior wall and right ventricle.

**Fig. 4:**
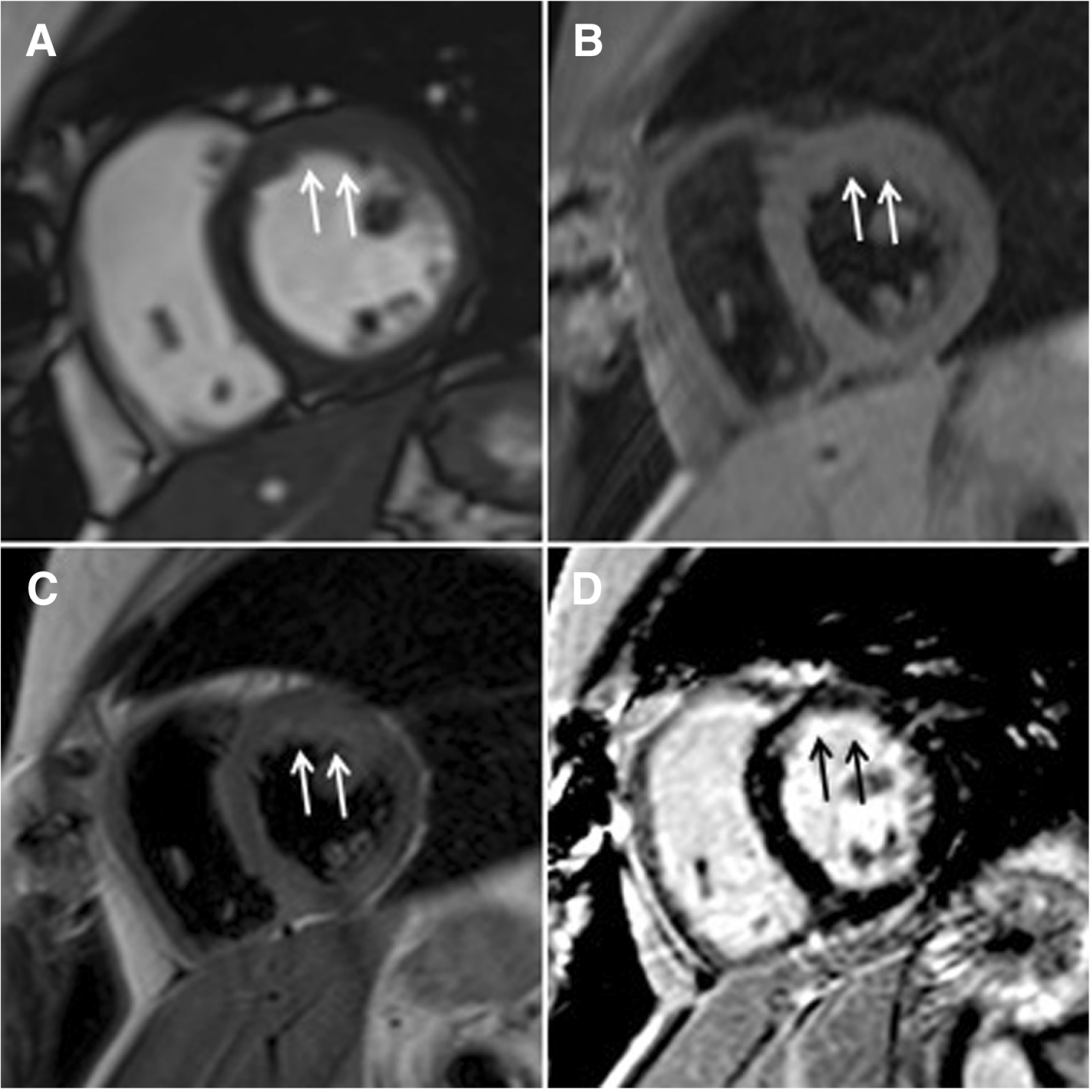
The CMR study after 7 cycles of chemotherapy treatment. Complete response in cine (**a**), T1-weighted (**b**), T2-weighted (**c**) and LGE (**d**) images. LGE = late gadolinium enhancement

Chemotherapy with doxorubicin and ifosfamide was initiated. The scheme was: ifosfamide 3 g/m^2^/day 1-3 days, Mesna 100% ifosfamide dose, doxorubicin 75 mg/m^2^/day 1 day. *Granulocyte-colony stimulating factors* (*GCSF*) were given. According to CTCAE (Common Terminology Criteria for Adverse Events) only I grade neutropenia and thrombocytopenia were observed. Totally, 7 cycles were completed (total cumulative doxorubicin dose 525 mg/m^2^). Echocardiography and CMR were performed to assess the results of chemotherapy treatment and revealed a complete response – only signs of fibrosis without any signs of tumor were visible in CMR images (Fig. 4). The chest and abdomen CT were repeated and did not demonstrate any findings suggesting metastasis.

**Fig. 5:**
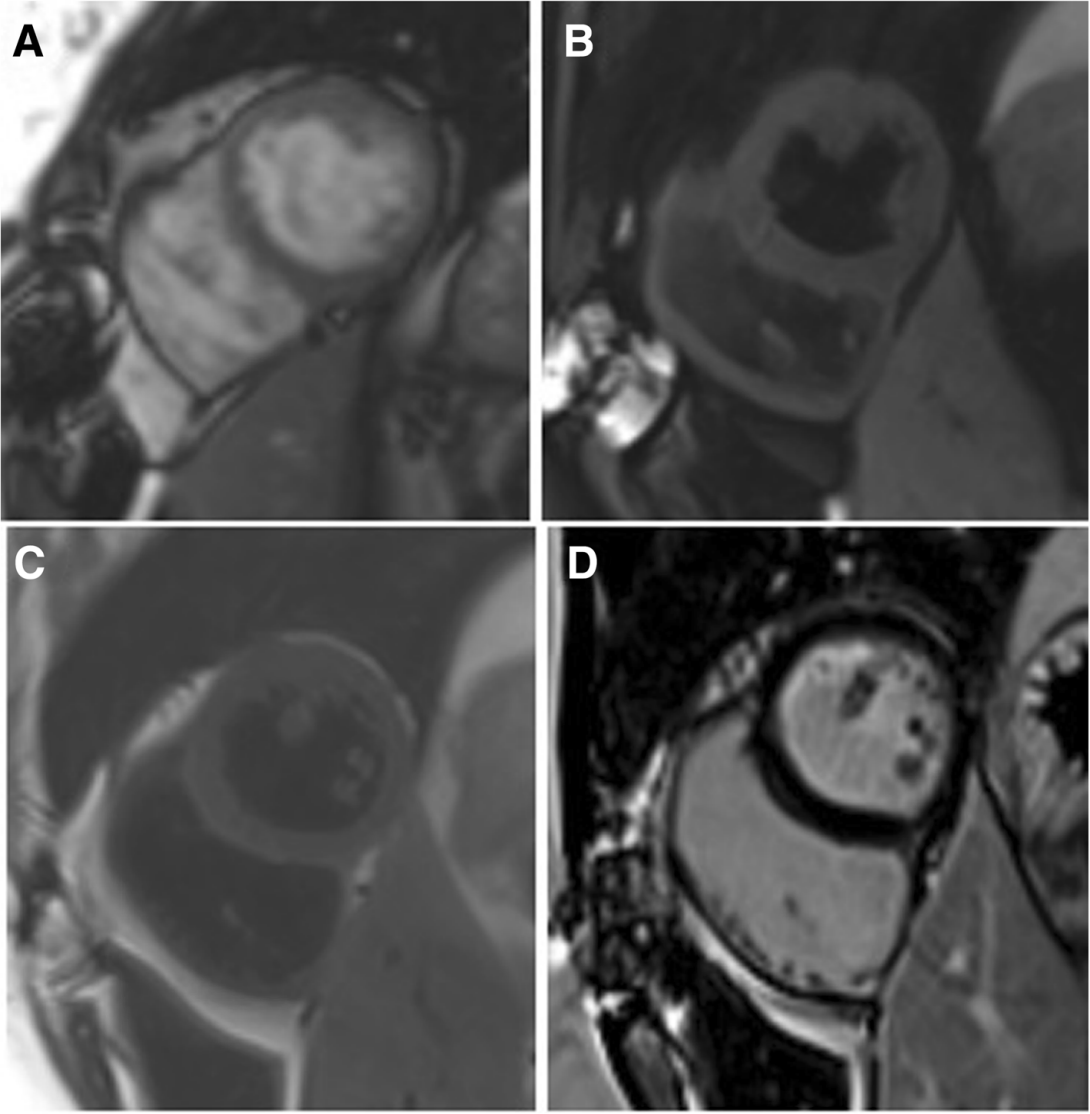
CMR after 26 months from the diagnosis. Still in complete response in cine (**a**), T1-weighted (**b**), T2-weighted (**c**) and LGE (**d**) images

Follow ups with TTE, CMR and chest, abdomen and pelvic CT is performed every 3 months. Twenty-six months from initial diagnosis the patient is still free of recurrence of tumor (Fig. 5) with no compromises of the quality of life.

## Discussion and conclusions

Primary cardiac tumor is a very rear pathology with an incidence of less than 0.1%. The incidence has increased over 3 eras (per 100 million persons): 25.1 in 1973 to 1989, 30.2 in 1990 to 1999, and 46.6 in 2000 to 2011. During those years the incidence of sarcomas and lymphomas increased and the incidence of mesotheliomas decreased [6, 7]. The patients can commonly present with dyspnea (48%) as noticed in the case of our patient, chest pain (22%), heart failure (13%), and pericarditis (5%) [8].

Most sarcomas grow very fast, and cause death through widespread infiltration of the myocardium, obstruction of blood flow through the heart, and/or distant metastases. Even with complete resection, most patients develop recurrent disease rapidly, thus according to different sources the median survival is typically 6 to 12 months [5]. The 32-year experience in Mayo clinic showed that the most common histological subtype of heart sarcomas was angiosarcoma (41%) and median survival with complete resection was 17 months compared to 6 months with incomplete resection [9]. Due to poor results, different experimental techniques are being used. In China, Shanghai, 6 patients with non-metastatic primary cardiac sarcoma underwent cardiac transplantation, they also included other 40 patients after cardiac transplantation of the same reason found in literature. Among the 46 patients overall median survival was 16 months, ranging from 2 to 112 months, with the worst results in angiosarcoma group (9 vs 36 months). The patients with grade 2 sarcomas survived much longer than with grade 3 tumors (85 vs. 18 months) [10]. Auto-transplantation of the heart in the aim to perform a complete resection has also been attempted. Survival up to 5.5 years after complete surgical resection with the auto-transplantation technique has been reported [11]. Another successful cardiac autotransplantation procedure in the case of spindle cell sarcoma was reported in Japan. The tumor was excised completely together with most of the left atrium, which was the reconstructed using bovine pericardial patch. The patient was doing well 30 months after surgery [12].

UPSs are aggressive and locally invasive tumors, frequently making complete surgical excision unfeasible, which leads to a poor prognosis and a low survival rate [4]. Recently a similar case of 61-year-old woman with undifferentiated pleomorphic sarcoma of the left atrium was reported. The resection was incomplete, the patient presented with a relapse 6 months later. The outcome was much different. Anthracycline-based palliative chemotherapy was started, despite that the patient died 9 months after initial diagnosis [13]. However, radical surgical treatment can be successful and lead to good survival rate, e.g.:78-year-old woman with the UPS in left atrium underwent radical surgical resection and refused adjuvant chemotherapy. She survived 3 years and then presented with advanced metastatic disease and decompensated heart failure and died [14].

Neoadjuvant or adjuvant chemotherapy has been used to improve outcomes of the poor results with resection alone. Adjuvant chemotherapy with doxorubicin and ifosfamide is usually recommended postoperatively [11]. A study in France was conducted where effects of chemotherapy which included doxorubicin after surgical resection was analyzed. Median survival of 15 patients was 12 months, survival rate of 2 years was 26%. Survival was significantly longer for patients with completely resected tumors (22 vs 7 months) and those who did not have angiosarcoma (18 vs 7 months). [15] Although all in all studies conclude that chemotherapy does not change the natural course of the disease there are a few cases in literature where multidisciplinary approach to different type primary cardiac sarcomas including chemotherapy has led to good survival rates [16–18]. A study from Cleveland Clinic where totally 42 patients with cardiac sarcoma treated from 1988 to 2013 were analyzed stated that multimodality therapy (any combination of surgery, radiation therapy, and chemotherapy) was associated with improved survival compared to surgery, radiation therapy of chemotherapy alone [3].

The case we presented is unique because without radical surgery and after chemotherapy the patient got into complete remission which lasts already 26 months. This already overcomes all median survival rates cited in literature even with the complete resection of tumor. We believe this is due to aggressive multidisciplinary approach.

The review is limited because the disease is very rare, the data are scarce, mostly case reports and experiences of single institutions are published. Therefore, individual approach to every case is extremely important and treatment options should be discussed thoroughly in the multidisciplinary team.

## Abbreviations

CMR: Cardiovascular magnetic resonance
CT: Computed tomography
CTCAE: Common Terminology Criteria for Adverse Events
GCSF: Granulocyte-colony stimulating factors
IAS: Interatrial septum
LA: Left atrium
LDH: Lactate dehydrogenase
LV: Left ventricle
TTE: Transthoracic echocardiography
UPS: Undifferentiated pleomorphic sarcoma
